# Speech‐language profiles in the context of cognitive and adaptive functioning in SATB2‐associated syndrome

**DOI:** 10.1111/gbb.12761

**Published:** 2021-09-13

**Authors:** Lot Snijders Blok, Y. Max Goosen, Leenke van Haaften, Karen van Hulst, Simon E. Fisher, Han G. Brunner, Jos I. M. Egger, Tjitske Kleefstra

**Affiliations:** ^1^ Human Genetics Radboud University Medical Center Nijmegen The Netherlands; ^2^ Language & Genetics Department Max Planck Institute for Psycholinguistics Nijmegen The Netherlands; ^3^ Donders Institute for Brain Cognition & Behaviour, Radboud University Medical Center Nijmegen The Netherlands; ^4^ Centre of Excellence for Neuropsychiatry Vincent van Gogh Institute for Psychiatry Venray The Netherlands; ^5^ Department of Rehabilitation Radboud University Medical Center Nijmegen The Netherlands; ^6^ Donders Institute for Brain, Cognition & Behaviour Centre for Neuroscience, Radboud University Nijmegen The Netherlands; ^7^ Department of Clinical Genetics MHeNS School of Neuroscience, and GROW‐School for Oncology and Developmental Biology, Maastricht University Medical Center Maastricht The Netherlands; ^8^ Stevig Specialized and Forensic Care for People with Intellectual Disabilities Dichterbij Oostrum The Netherlands; ^9^ Donders Institute for Brain, Cognition & Behaviour Centre for Cognition, Radboud University Nijmegen The Netherlands

**Keywords:** clinical management, communication disorder, contextual neuropsychology, neurodevelopmental disorder, SATB2 associated syndrome, speech and language impairments

## Abstract

*SATB2*‐associated syndrome (SAS) is a neurodevelopmental disorder caused by heterozygous pathogenic variants in the *SATB2* gene, and is typically characterized by intellectual disability and severely impaired communication skills. The goal of this study was to contribute to the understanding of speech and language impairments in SAS, in the context of general developmental skills and cognitive and adaptive functioning. We performed detailed oral motor, speech and language profiling in combination with neuropsychological assessments in 23 individuals with a molecularly confirmed SAS diagnosis: 11 primarily verbal individuals and 12 primarily nonverbal individuals, independent of their ages. All individuals had severe receptive language delays. For all verbal individuals, we were able to define underlying speech conditions. While childhood apraxia of speech was most prevalent, oral motor problems appeared frequent as well and were more present in the nonverbal group than in the verbal group. For seven individuals, age‐appropriate Wechsler indices could be derived, showing that the level of intellectual functioning of these individuals varied from moderate–mild ID to mild ID‐borderline intellectual functioning. Assessments of adaptive functioning with the Vineland Screener showed relatively high scores on the domain “daily functioning” and relatively low scores on the domain “communication” in most individuals. Altogether, this study provides a detailed delineation of oral motor, speech and language skills and neuropsychological functioning in individuals with SAS, and can provide families and caregivers with information to guide diagnosis, management and treatment approaches.

## Graphical abstract

This study provides a detailed delineation of oral motor, speech and language skills and neuropsychological functioning in individuals with SATB2 associated syndrome.

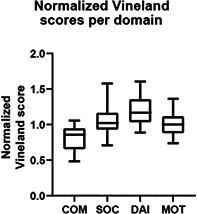

## 1 INTRODUCTION

The introduction of new DNA sequencing technologies (next‐generation sequencing) has rapidly improved the identification of genes of which high‐penetrance disruptive variants can cause neurodevelopmental disorders. Amongst the most commonly affected genes in neurodevelopmental disorders is *SATB2*.^1^ The neurodevelopmental disorder associated with pathogenic variants in this gene is known as *SATB2*‐associated syndrome (SAS). The exact prevalence of SAS is not known. However, the yield after applying exome sequencing in a large cohort of individuals with undiagnosed developmental disorders showed the frequency of pathogenic variants in *SATB2* to be 0.3% (14/4294 probands).^1^

SAS presents with marked craniofacial dysmorphisms, intellectual disability (ID), developmental delay, as well as generally restricted or absent speech and severely impaired communicative skills.^2^ Individuals with SAS often use communication methods other than (or in addition to) spoken language, such as gestures, sign language and/or augmentative and alternative communication (AAC) devices. In addition to speech problems, other features related to oral motor skills or oral abnormalities are common, including cleft palate, teeth anomalies, drooling and feeding problems.^2^

SAS is caused by heterozygous disruptions of the *SATB2* gene. These are mostly variants with a clear loss‐of‐function effect (frameshift and nonsense variants), but missense variants, variants predicted to affect splicing and copy number variants are reported as well.^3^ The SATB2 protein is a transcription factor with important roles in cortical development.^4^ One could hypothesize that loss‐of‐function of *SATB2* might disproportionately affect the development of higher cognitive functions, such as attention, memory and executive functioning. While speech problems are prominent in SAS, there is limited information about mechanisms underlying the oral motor, speech and language impairments observed in affected individuals, other than one recent study on the assessment of speech and language phenotypes in a SAS cohort.^5^ That study found that individuals with SAS generally show prominent language impairments, childhood apraxia of speech and various oral motor problems, including hypernasal resonance, pharyngeal phase dysphagia and drooling.^5^

The current study aimed to contribute to the understanding of speech and language abnormalities in SAS in the context of general developmental capacities and cognitive and adaptive functioning. The study design included a detailed characterization of oral motor, speech and language profiles combined with neuropsychological testing in 23 individuals with a molecularly confirmed diagnosis of SAS.

## 2 METHODS

### 2.1 General study design and data collection

#### 2.1.1 Study design

This study has an observational and cross‐sectional study design and was approved by the medical research and ethics committee Arnhem‐Nijmegen (CMO Arnhem‐Nijmegen; study number NL64562.091.18). All study procedures were in line with the principles of the Declaration of Helsinki. Recruitment and inclusion for the study took place between April and November 2019. After inclusion and the informed consent procedure, individuals were invited for two testing visits within the Radboud University Medical Center in Nijmegen, the Netherlands: one visit with one of the two speech‐language therapists (SLTs), and one visit with a healthcare psychologist. During one of these two visits, a clinical geneticist in training collected details on medical history and growth parameters. In addition to this, parents and/or caregivers were asked to fill in standardized questionnaires about the patient with SAS. Data collection finished in March 2020.

#### 2.1.2 Individuals

Individuals with SAS from the Netherlands and Belgium were recruited via the Dutch SAS family support group or via the Clinical Genetics department where their SAS diagnosis was established. In order to be eligible to participate in the study, individuals had to meet all the following three criteria: (a) established molecular diagnosis of *SATB2*‐associated syndrome, (b) age of at least 2 years old at time of testing and (c) raised in a Dutch‐speaking family with Dutch as first language. There was one exclusion criterion: Individuals with SAS who also had another molecular diagnosis that likely contributed to their developmental phenotype were excluded from participation, for example, individuals with larger copy number variants not only affecting *SATB2* but also encompassing additional neurodevelopmental disorder‐associated genes. In total, 23 individuals were included for participation in the study.

#### 2.1.3 General data collection

Data on developmental and medical history were collected via medical file notes and a standardized medical history during one of the visits. Growth parameters were measured during the visit or, if this was not possible, derived from recent measurements in another context. All official molecular test reports with the *SATB2* diagnosis were collected, and variant details were converted into standardized nomenclature using hg19 as a reference genome and NM_001172509.1 (*SATB2* isoform 1) as the standard transcript. All data were de‐identified and stored in a secure and study‐specific Castor EDC database.^6^

### 2.2 Speech and language profiling

#### 2.2.1 Communication measures

Contingent upon the use of words and dominant communication mode, individuals were categorized as primarily nonverbal (an expressive vocabulary of no more than 10 words, communicating nonverbal more than verbal) or verbal (an expressive vocabulary of more than 10 words with speaking as the primary mode of communication).

The communication function classification system (CFCS)^7^ was used to rate overall communication abilities. The CFCS is a validated discriminative tool that allows clinicians and parents to categorize children's communication skills into five mutually exclusive levels (CFCS I‐V) of everyday communicative function with sending and receiving messages via any modality (e.g., spoken language, sign language, speech‐generating electronic devices) with familiar and unfamiliar communication partners.

Utilized forms of augmentative and alternative communication (AAC) were recorded and categorized in (a) unaided—no‐tech (gestures, manual signs, facial expressions, vocalizations, verbalizations, body language), (b) aided—low‐/light‐tech (pictures, objects, photographs, writing, communication boards/books) and (c) aided—high‐tech (speech generating devices [SGD], single‐message devices and recordable/digitized devices, AAC software that enables dynamic symbol/language representation and that is used with some form of technology hardware such as computer, tablet, or smartphone).^8^

#### 2.2.2 Language measures

Receptive vocabulary was assessed in most individuals with the Dutch version of the Peabody Picture Vocabulary test‐III,^9^ yielding a vocabulary quotient. The Schlichting tests for language comprehension and language production^10^ were used to measure receptive and expressive language skills. These norm‐based standard scores or *Q* scores have a mean score of 100 (SD 15), with a score of 85–115 representing average range performance.

When the administration of the Schlichting tests was not possible due limited language and/or understanding, the Dutch Nonspeech Test (NNST)^11^ was used. This test comprises a receptive scale and an expressive scale. Scores on both scales were expressed in percentile scores, with a mean score of 50.

Subtests of the Dutch version of the clinical evaluation of language fundamentals (CELF)^12^ were used instead of the Schlichting tests when individuals had a sufficient level of language. The subtests “concepts and following directions,” “expressive vocabulary,” “recalling sentences,” and “formulating sentences” were administered.

The Q scores and percentile scores of all the language assessments were interpreted as mild (1–1.5 *SD* below mean), moderate (1.5–2 *SD* below mean) and severe (>2 *SD* below mean).

#### 2.2.3 Speech measures

Where children had sufficient speech, a conversational sample was obtained. The observed speech symptoms provided a basis to form a clinical impression of characteristics of different speech disorders, including a phonological delay or disorder, childhood apraxia of speech (CAS), dysarthria, or an articulation deficit. Speech characteristics were analyzed using Dodd's Model for Differential Diagnosis^13^ and protocols for the classification of dysarthria.^14^

The intelligibility of speech was measured in primarily verbal individuals using the Dutch version of the intelligibility in context scale (ICS).^15^ This seven‐item questionnaire rates the degree to which the patient's speech is understood by different communication partners (parents/life partners, immediate family, extended family, friends, acquaintances, teachers/colleagues, strangers) on a five‐point scale (1 = never, 2 = rarely, 3 = sometimes, 4 = usually, 5 = always).

#### 2.2.4 Feeding and oral motor evaluation

A specifically designed questionnaire for problems with swallowing related to different consistencies of food was used in all individuals. It also included questions regarding drooling and dental problems. This semi‐structured questionnaire is used in earlier studies where it has demonstrated its usefulness and importance to differentiate dysphagia characteristics.^16^, ^17^ Problems with only chewing (refers to problems in the oral phase) and chewing and choking (refers to problems in the oropharyngeal phase) were scored with a five‐point scale and recoded into two categories (−) no problems or (+) problems to a certain extent (2 = less than once a day, 3 = once every day, 4 = several times a day, 5 = food is not offered).

Structural or functional impairments of the oral region were assessed with the self‐composed oral‐facial motor assessment for children (OMAC). This assessment tool examines oral motor function (e.g., face, lips, tongue, velum, jaw), oral‐facial structural integrity (e.g., symmetry, lip seal), strength (e.g., eye closure, lip closure, tongue, jaw) and the saliva swallow (e.g., slurping, swallowing on demand) by observation. Problems with the performance or imitation of the items were scored and recoded in the category (−) no problems and (+) problems to a certain extent.

### 2.3 Neuropsychological assessment

#### 2.3.1 Intellectual and cognitive functioning

For the reliable and valid assessment of intellectual functioning, three Dutch‐language variants of the Wechsler intelligence scales were used, depending on the age of the individual. The Wechsler preschool and primary scale of intelligence third edition (WPPSI‐III‐NL^18^) was used for individuals aged between 2;6 and 7;11 years, the Wechsler Intelligence Scale for Children Fifth Edition (WISC‐V‐NL^19^) for individuals with chronological ages between 8 and 17;11 years and the Wechsler Adult Intelligence Scale Fourth Edition (WAIS‐IV‐NL^20^) for individuals of 18 years and older. The WPPSI‐III‐NL, WISC‐V‐NL and WAIS‐IV‐NL provide a full scale IQ (FSIQ, *M* = 100, *SD* = 15), based on the performance on four (age group 2;6–3;11), seven (age group 4–7;11) and 10 subtests, respectively (WISC‐V‐NL age range 6–16;11, WAIS‐IV‐NL age range 16–84;11). Raw scores are converted to Wechsler standard scores (range 1–19) which are used to calculate IQ and index scores. In addition to Full Scale IQ, the WPPSI‐III‐NL provides a Verbal IQ (VIQ), a Performance IQ (PIQ) and a Processing Speed Quotient (PSQ; only for the age group 4–7;11). The WISC‐V‐NL provides a Verbal Comprehension Index (VCI), Visual Spatial Index (VSI) and indices for Fluid Reasoning (FRI), working memory (WMI) and processing speed (PSI). The WAIS‐IV‐NL provides indices for Verbal Comprehension (VCI), Perceptual Reasoning (PRI), Working Memory (WMI) and Processing Speed (PSI). When age appropriate testing was not possible due to limited language and/or understanding, the WPPSI‐III‐NL was administered. Raw scores were converted into developmental age equivalents ranging from “below 2;7” to “above 7;10.” Although test administration was performed according to standard procedures, slight alterations were made to compensate for language problems of the individuals. For instance, individuals were allowed to respond using Dutch Sign Language and/or using AAC when verbal responses were required and extra verbal cues and explanation were given to engage individuals further when non‐compliant (i.e., “testing the limits”).

#### 2.3.2 Adaptive functioning

Adaptive behavior has been described as the combination of conceptual, social and practical skills acquired to function adequately in daily life.^21^ The level of adaptive functioning was measured using the Vineland Screener 0–6 years,^22^ filled out by parents. This questionnaire is a Dutch screener version of the gold standard Vineland adaptive behavior scales^23^ and consists of 72 questions, providing a total score and four domain scores: communication, social functioning, daily functioning and motor skills. Raw scores were converted to developmental age scores (in months), reflecting the level of adaptive functioning.^22^

To enable inter‐individual comparison of Vineland profiles, individual Vineland scores (age equivalents) per domain were normalized by dividing each score by the total Vineland score (age equivalent) of the same individual. A normalized score of 1.0 indicates that the age equivalent of the domain score is similar to the age equivalent of the total score of this individual.

#### 2.3.3 Behavioral problems

The presence of behavioral problems was measured by parent‐based reports, using age‐specific versions of the Achenbach system of empirically based assessment^24^: the Dutch versions of the child behavior checklist (CBCL/1,5–5^25^ and CBCL/6–18^26^) and the proxy version of the adult behavior checklist (ABCL/18–59).^27^ These parent‐based questionnaires consist of 100, 113 and 134 items, respectively and provide a total score for observed behavioral problems, scales for internalizing (i.e., anxiety, depression and withdrawal) and externalizing (i.e., aggressive behavior, conflict with others/social mores) problems and several syndrome subscales. In this study, only the syndrome scales were included that were present in all three versions: somatic, anxious, withdrawn, attention and aggression problems. Raw scores were converted to standardized T‐scores. For the total score and internalizing and externalizing scales, a score of 64 and higher is considered to be in the clinical range (i.e., consideration of professional help is warranted), for the syndrome scale the cut‐off for a score in the clinical range is a T score of 70.^25^, ^26^, ^27^

## 3 RESULTS

### 3.1 Individuals and characteristics

In total, 32 individuals were examined for eligibility to participate in the study. Nine were not included, because the parents/caregivers decided not to participate after being informed about study details (*n* = 6), because the child was not raised with Dutch as first language (*n* = 2) or because the *SATB2* disruption was part of a large microdeletion with many other genes possibly affecting neurodevelopment (*n* = 1). A total of 23 individuals started participation in the study, all of whom completed it; 70% of these individuals were male. The age of individuals at inclusion varied from 2;10 to 40;8 years old (median age 11;7). Growth parameters and other baseline characteristics are included in Table 1.

**TABLE 1:** Patient characteristics

	Nonverbal (*n* = 12)	Verbal (*n* = 11)	Total (*n* = 23)
**General**			
Gender (% male/% female)	67%/33%	73%/27%	70%/30%
Median age at inclusion in y;m (range, IQR)	10;10 (2;10–39;3, 13;5)	11;7 (5;6–40;8, 18;4)	11;7 (2;10–40;8, 15;7)
**Genetic diagnosis**			
SNV	92%	91%	91%
nonsense	50%	18%	35%
frameshift	25%	36%	30%
missense	8%	27%	17%
splice	8%	9%	9%
CNV	8%	9%	9%
Confirmed de novo	83%	100%	91%
Mosaic variant in individual	0%	9%	4%
Median age of molecular diagnosis in y;m (range, IQR)	8;1 (0;5–38;11, 11;10)	10;10 (4;0–37;7, 14;7)	10;10 (0;5–38;11, 12;6)
**Growth parameters**			
Mean birth weight (*SD*)	3570 g (446)	3485 g (626)	3531 g (524)
Mean height corrected for age (*SD*)	−0.3 *SD* (1.5)	0.8 *SD* (1.3)	+0.3 *SD* (1.4)
Mean weight corrected for age (*SD*)	−0.3 *SD* (1.3)	−0.5 *SD* (1.4)	−0.4 *SD* (1.4)
Mean head circumference corrected for age (*SD*)	0.0 *SD* (0.7)	0.2 *SD* (0.8)	0.0 *SD* (0.8)
**Neuro/development**			
Median age of walking in months (range, IQR)	23 (18–42, 6)	23.5 (13.5–36, 9)	23 (13.5–42, 8)
Gross motor delays	100%	82%	91%
Fine motor delays	100%	100%	100%
Epilepsy (confirmed)	17%	9%	13%
**Other**			
Cleft palate	50%	18%	35%
Dental problems	83%	91%	87%
Vision problems	50%	36%	43%
Hearing loss	0%	0%	0%

Details on the *SATB2* variants in the individuals are included in Table S1. In short, the majority of individuals (21/23; 91%) had a heterozygous single nucleotide variant (SNV) affecting *SATB2*; two individuals (9%) had a de novo 2q33.1 microdeletion (Table 1). Almost all variants (21/23; 91%) were confirmed to be de novo, hence not present in blood‐derived DNA of either of the two parents of the individuals. Two individuals were siblings and carried the same de novo variant, suggesting germline mosaicism in one of the parents. Constitutive mosaicism was not detectable by Sanger sequencing of parental blood samples. In one individual, the *SATB2* variant was found to be a mosaic variant and present in 32 of 143 exome sequencing reads (~22%). The age at which the molecular diagnosis of *SATB2* was established in each individual varied between 0;5 years and 38;11 years, with a median of 10;10 years (Table 1).

## 4 COMMUNICATION

Verbal communication was primarily used by 11 individuals (47.8%), whereas 12 individuals were nonverbal (52.2%). As a group, individuals with primarily verbal communication and nonverbal individuals were comparable in terms of chronological age: median age of the verbal group was 11;7 years (range 5;6–40;8 years) and that of the nonverbal group was 10;10 years (range 2;10–39;3).

AAC was used by most individuals (*n* = 20/23; 87.0%). The most commonly used form of AAC was signed (*n* = 14/23; 60.87%). Signs were used alone or in combination with other forms of unaided or aided AAC, for example, vocalizations, gestures, objects, pictures/photographs, communication books, AAC software and speech‐generating devices.

**FIGURE 1:**
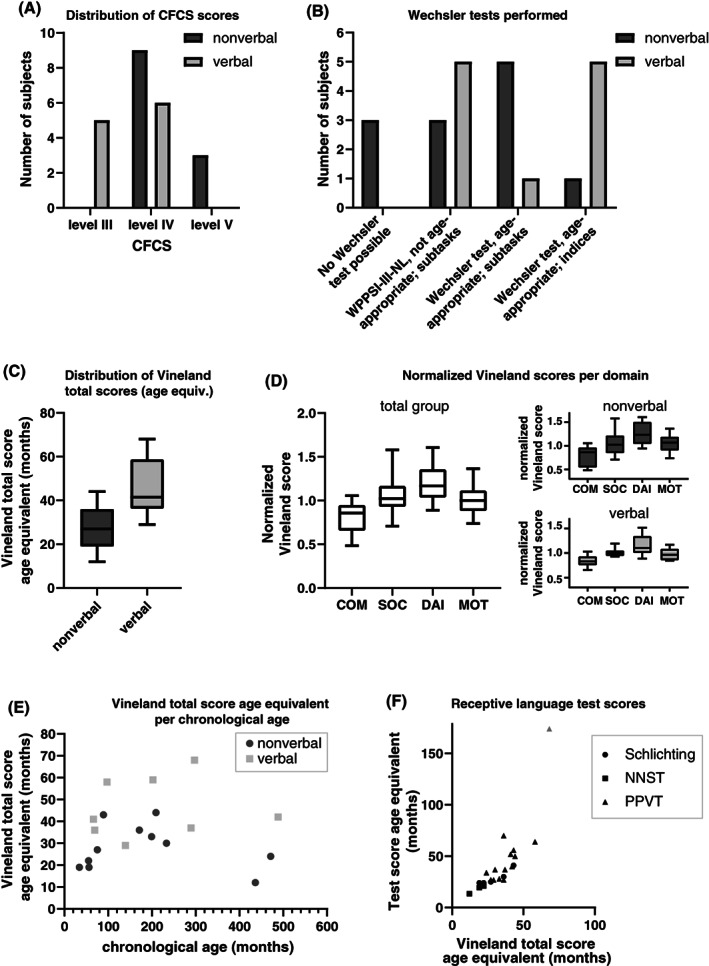
Visual summary of test results. (A) Distribution of CFCS test scores in nonverbal and verbal individuals. (B) Wechsler tests and subtests performed in nonverbal and verbal individuals. (C) Distribution of age equivalents of Vineland total scores in nonverbal and verbal individuals. (D) Distribution of normalized Vineland scores for each of the four Vineland domains. Individual Vineland scores (age equivalents) per domain were normalized by dividing each score by the total Vineland score (age equivalent) of the same individual: a score of 1.0 indicates that the age equivalent of the domain score is similar to the age equivalent of the total score of this individual. (E) Age equivalents of Vineland total scores (months) obtained in nonverbal and verbal individuals versus chronological age (months). (F) Age equivalents of test scores of three different receptive language (sub)tests, compared with Vineland total score age equivalents. The gray triangle indicates a ceiling effect for the Vineland test score, as the maximum score of 68 months was obtained for this test

On the CFCS, all individuals exhibited problems with reliable communication with unfamiliar partners (CFCS level III, IV, or V). Three individuals (13%) were rated level V (seldom effective sender and receiver even with familiar partners), 15 individuals (65%) level IV (sometimes effective sender and receiver with familiar partners) and 5 individuals (22%) level III (effective sender and receiver with familiar partners). In the verbal group, all individuals were rated with level III or IV and in the nonverbal group all individuals had level IV or V (Figure 1A).

### 4.1 Language

Receptive language abilities were measured in 21 individuals. Two individuals were not assessed because test procedures were not developmentally appropriate or individuals were not able to be tested. All the tested individuals showed severe receptive language deficits when compared with age‐related peers, except for one (individual 5) with a mild deficit. Expressive language could be measured in nine verbal individuals. Eight of them had a severe expressive language deficit and one had a moderate to severe deficit (individual 10) when compared with age‐related peers.

### 4.2 Speech

All individuals, except one, showed difficulties with speech production. For the 10 remaining verbal individuals a speech diagnosis could be established. The 12 nonverbal individuals did not produce enough verbal utterances to be able to differentiate between speech diagnoses although speech symptoms could be described. Six of these 12 individuals had no verbal utterances. The other six had an expressive vocabulary of fewer than 10 words; three of those individuals showed symptoms of phonological delay, and one had symptoms of dysarthria. In the verbal group, the most common speech diagnosis was Childhood Apraxia of Speech (CAS) (*n* = 8). Two of these individuals showed symptoms of CAS only, while the other six showed symptoms of CAS combined with additional speech diagnoses; CAS and phonological delay (*n* = 2/6), CAS and dysarthria (*n* = 1/6), CAS and phonological delay and dysarthria (*n* = 3/6). The described symptoms of CAS were: words are pronounced sound by sound, fluency difficulties, problems with “automation” of words, difficulties with speaking on demand, difficulties with maximum repetition rate or diadochokineses. Dysarthria was characterized by slow speech, low pitch, hypernasality and difficulties with respiratory and voice coordination. One verbal individual showed symptoms of dysarthria only and another verbal individual showed symptoms of a phonological delay (i.e., delayed and atypical phonological speech‐sound processes) in combination with an articulation deficit (phonetic distortion). A single verbal individual had no characteristics of any speech disorder. For eight verbal individuals the ICS questionnaire was completed. The mean intelligibility score was 3.41 (range: 2.1–5). These findings indicate that the primarily verbal individuals in our study population are “sometimes” to “usually” understand.

### 4.3 Feeding and oral motor evaluation

Feeding and swallowing problems were common in the total group of individuals with 87% affected (*n* = 20), while in the remaining three individuals no feeding problems were mentioned. In the nonverbal group, all individuals had feeding problems. In the verbal group, 80% exhibited feeding problems. For the feeding problems in the nonverbal group, 25% involved swallowing problems in the oral phase (e.g., chewing problems and overstuffing) and 75% involved the oropharyngeal phase (e.g., choking, aspiration). This is in contrast to the swallowing problems in the verbal group where 87.5% suffered from oral phase problems and only 12.5% showed oropharyngeal phase problems.

Almost half (48%, *n* = 11) of all 23 individuals showed problems with saliva control (drooling). In the nonverbal group (*n* = 12) there were more individuals suffering from drooling (67%, *n* = 8) compared with the verbal group (30%, *n* = 3). It was difficult for the individuals to collect saliva consciously and swallow it on request, only three individuals of the total group (all part of the verbal group) were able to slurp saliva and swallow it on demand.

Although data collection was not complete because of limited developmental capacities and cooperation of the individuals, oral motor functioning (movements of the face, lips, tongue, velum, jaw) was problematic for almost all individuals in the total group, except for two individuals in the verbal group. Oral facial structural integrity was normal in only three individuals in the nonverbal group, in contrast to five individuals in the verbal group. Only two individuals in the verbal group were able to generate strength when executing orofacial movements.

### 4.4 Neuropsychological functioning

Observation of behavior during testing procedures showed clear differences with regard to task understanding and concentration, both of which likely mediated task compliance that was further hampered in case of increased restlessness. We classified the results of formal neuropsychological testing using Wechsler scales in three groups (Figure 1B): a group in which age appropriate administration of Wechsler subtasks was possible (12 individuals), a group with results of non‐age appropriate administration of WPPSI‐III‐NL with individuals 8 years and older (eight individuals) and a group in which no formal testing was possible (three individuals).

The first group of individuals consisted of 12 individuals (52%) in which standardized Wechsler scores were derived. In four of these individuals, a complete profile could be established, with notable differences in indices. In three individuals, a single Wechsler Index score based on only non‐verbal tasks could be calculated. Based upon the different indices, the level of intellectual functioning in these seven individuals could be classified as moderate to mild ID (*n* = 1), mild ID (*n* = 5) and mild ID to below average (*n* = 1), respectively. In the remaining five individuals, the administered single subtasks were not sufficient to extract indices. Looking at the group of 12 individuals with standardized Wechsler scores, six individuals were classified as verbal and six were classified as nonverbal.

In the second group, consisting of eight individuals (35%), non‐age matched Wechsler administration of several subtasks of the WPPSI‐II‐NL were derived. These eight individuals had a chronological age of 9;3 to 40;8 and age equivalents calculated based on Wechsler subtask scores ranged from <2.7 to <7.1 years. Of these eight individuals, five were classified as verbal and three as nonverbal (Figure 1B; Table 2).

**TABLE 2:** Results per individual

Individual	Age at inclusion (years; months)	Type of *SATB2* variant^a^	Gross motor delays	Fine motor delays	Epilepsy	Vision problems	Cleft palate	Hearing loss	Verbal / Non‐verbal	CFCS level	Receptive language impairment	Expressive language impairment	Childhood Apraxia of Speech	Dysarthria	Phonological impairment
1	2;10	TV	+	+	−	−	−	−	NV	5	Severe	Severe	NA	NA	+
2	4;7	TV	+	+	−	+	−	−	NV	4	Severe	Severe	NA	NA	NA
3	4;8	TV	+	+	−	+	−	−	NV	4	Severe	NA	NA	NA	NA
4	5;4	MD	+	+	−	−	+	−	NV	5	NA	NA	NA	+	NA
5	5;6	mTV	+	+	−	−	−	−	V	4	Mild	Severe	+	−	+
6	5;9	MV	+	+	+	−	−	−	V	3	Severe	Severe	+	−	+
7	5;9	TV	+	+	−	−	−	−	V	3	Severe	Severe	+	−	+
8	6;3	TV	+	+	−	−	+	−	NV	4	Severe	Severe	NA	NA	+
9	7;5	MV	+	+	+	+	−	−	NV	4	Severe	NA	NA	+	NA
10	8;1	MV	−	+	−	−	−	−	V	3	Severe	Severe	−	−	−
11	9;3	TV	+	+	−	+	−	−	V	4	Severe	NA	+	−	−
12	11;7	TV	+	+	−	+	−	−	V	3	Severe	NA	−	−	+
13	14;3	SV	+	+	+	−	−	−	NV	4	Severe	NA	NA	NA	+
14	16;7	TV	+	+	−	+	+	−	NV	4	Severe	NA	NA	NA	NA
15	16;10	MV	+	+	−	−	−	−	V	4	MD	Severe	+	+	−
16	17;5	TV	+	+	−	+	+	−	NV	4	Severe	NA	NA	+	NA
17	19;5	TV	+	+	−	−	+	−	NV	4	Severe	NA	NA	NA	NA
18	21;4	TV	+	+	−	−	−	−	V	4	Severe	NA	+	+	+
19	24;1	SV	+	+	−	−	+	−	V	4	Severe	NA	+	+	+
20	24;9	MD	−	+	−	+	+	−	V	4	Severe	NA	−	+	−
21	36;4	TV	+	+	−	+	−	−	NV	5	Severe	Severe	NA	NA	NA
22	39;3	TV	+	+	−	−	+	−	NV	4	Severe	NA	NA	NA	NA
23	40;8	TV	+	+	−	+	−	−	V	3	Severe	NA	+	+	+

Individual	Articulation impairment	Feeding difficulties	Problems with oral motor function	Problems with oral facial structural integrity	Problems with oral motor strength	Problems with saliva swallow	Drooling	Wechsler test administered	Wechsler indices^b^	Vineland ‐ Communication skills^c^	Vineland ‐ Social skills^c^	Vineland ‐ Daily functioning skills^c^	Vineland ‐ Motor skills^c^	Vineland ‐ Total score^c^	T‐scores in clinical range^d^
1	NA	+	NA	NA	NA	NA	+	WPPSI‐III‐NL		18	30	21	14	19	−
2	NA	+	NA	NA	NA	NA	+	WPPSI‐III‐NL		19	19	23	30	22	1,2,3,4,5,6
3	NA	+	NA	NA	NA	NA	+	WPPSI‐III‐NL		19	23	18	20	19	1,2,3,4,5
4	NA	+	NA	NA	NA	NA	+	NA		MD	MD	MD	MD	MD	MD
5	−	+	+	NA	NA	NA	+	WPPSI‐III‐NL	VIQ 55, PIQ 67, PSI 59, FSIQ 55	31	39	46	48	41	−
6	−	+	NA	NA	NA	NA	+	WPPSI‐II–NL	PIQ 55	29	43	32	40	36	−
7	−	+	NA	NA	NA	NA	+	WPPSI‐III‐NL		MD	MD	MD	MD	MD	−
8	NA	+	+	−	+	+	+	WPPSI‐III‐NL		19	23	35	32	27	−
9	NA	+	+	NA	NA	NA	−	WPPSI‐III‐NL	PIQ 55	24	44	53	48	43	−
10	−	−	−	−	−	−	−	WISC‐V‐NL	VCI 70, VSI 67, FRI 69, WMI 59, FSIQ 54	50	61	60	58	58	−
11	−	−	NA	NA	NA	NA	−	WPPSI‐III‐NL^e^		MD	MD	MD	MD	MD	MD
12	+	+	+	−	+	+	−	WPPSI‐III‐NL^e^		19	28	44	27	29	1,3,5
13	NA	+	+	−	+	+	−	WPPSI‐III‐NL^e^		38	48	34	27	36	1,4
14	NA	+	+	−	+	+	−	WAIS‐IV‐NL		16	35	53	30	33	−
15	−	+	+	−	+	−	−	WISC‐V‐NL	VCI 68, VSI 45, FRI 61, WMI 55, PSI 56, FSIQ 50	56	61	64	50	59	1,2
16	NA	+	+	+	NA	NA	+	WPPSI‐III‐NL^e^		24	44	59	47	44	−
17	NA	+	+	+	NA	NA	+	WPPSI‐III‐NL^e^		16	24	48	33	30	MD
18	−	−	+	−	+	NA	−	WPPSI‐III‐NL^e^		MD	MD	MD	MD	MD	MD
19	−	+	+	+	NA	NA	−	WPPSI‐III‐NL^e^		28	35	48	37	37	−
20	−	+	−	−	−	−	−	WAIS‐IV‐NL	VCI 81, PRI 64, WMI 55, PSI 84, FSIQ 67	70	70	68	58	68	−
21	NA	+	NA	NA	NA	NA	+	NA		11	14	18	11	12	1,2,3,4,5,7
22	NA	+	NA	NA	NA	NA	−	NA		23	17	28	29	24	3
23	−	+	+	+	NA	+	−	WPPSI‐III‐NL^e^		36	39	57	37	42	−

*Note*: + = present, − = not present, NA = not assessed as not developmentally appropriate or not able to be tested, MD = missing data.

^a^ TV = truncating variant, MV = missense variant, SV = splice variant, MD = microdeletion, mTV = mosaic truncating variant.

^b^ VCI = Verbal Competency Index, VSI=Visual Spatial Index, FRI = Fluid Reasoning Index, PRI = Perceptual Reasoning Index, VIQ = Verbal IQ, PIQ = Performal IQ, WMI = Working Memory Index, PSI = Processing Speed Index, FSIQ = Full Scale IQ.

^c^ Age equivalent in months.

^d^1 = Total, 2 = Internalizing, 3 = Externalizing, 4 = Syndrome scale Attention, 5 = Syndrome scale Aggression, 6 = Syndrome scale Withdrawing, 7 = Syndrome scale Somatic.

^e^ Non age‐matched administration.

The last group consists of three individuals (13%), who were noneligible for testing in either form, because of a lack of understanding and cooperation. These three individuals were all classified as nonverbal.

As measured by the Vineland Total score (*n* = 19), a distinction between chronological and developmental age ranges was found: 35–489 months versus 12–68 months, respectively (Figure 1E). One individual (individual 20, 24 years old) obtained the maximum score of 68 months on the Vineland Screener (i.e., representing a ceiling effect), resulting in scores which do not reflect the actual (higher) level of adaptive functioning. When excluding this single case, the highest adaptive functioning score is 59 months. Inspection of the normalized age equivalents for the total group of individuals showed distinct differences in the domain profile, where the level of daily functioning appeared to be relatively high and the level of communication skills relatively low compared with the total score (Figure 1D). When distinguishing between verbal and nonverbal individuals, identical patterns were seen across subdomains (Figure 1D). The overall levels of adaptive functioning seem to be higher in the verbal group compared with the nonverbal group (Figure 1C).

When comparing the results of receptive language tests (converted to age equivalents) with the age equivalents matching the Vineland adaptive functioning total score, the results of these language tests seem to align with the estimated level of adaptive functioning (Figure 1F).

By reviewing clinical histories with parents and caregivers, sleep disturbances were mentioned in 13 individuals, varying from trouble falling asleep and difficulty staying asleep to increased mobility and/or anxiety. When asked about possible sensory processing problems, these were mentioned in 15 individuals (e.g., high pain threshold, easily overstimulated). Present challenging behaviors were mentioned for five individuals, whereas in a sixth individual these problems had occurred earlier. Regarding psychiatric comorbidity, in three individuals concentration problems were mentioned, and in four individuals autistic traits were mentioned, without meeting formal criteria for a classification of autism spectrum disorder.

Based on CBCL/ABCL total (t‐)scores (*n* = 18), behavioral problems within the clinical range were reported in six individuals (33%) of which four are classified as non‐verbal and two as verbal. In three individuals (16%, all nonverbal) both internalizing and externalizing problems were reported. In two individuals (11%, one verbal, one nonverbal) only externalizing problems were reported and in one individual (5%, verbal) only internalizing problems. Three individuals (22%, all nonverbal) scored within the clinical range for both attention and aggression problems, of which one (5%) scored within the clinical range on somatic problems, and one on withdrawn behavior (5%). One individual (5%, nonverbal) scored within the clinical range for attention problems and one (5%, verbal) for aggression problems (Table 2).

### 4.5 Genotype–phenotype comparison

In terms of genotype–phenotype relations, we looked more specifically to the different types of genetic variants disrupting *SATB2* that were present in our cohort and the associated general developmental and speech‐language phenotype. Fourteen individuals had a nonsense or frameshift variant likely causing haploinsufficiency via nonsense‐mediated mRNA decay. Two individuals had a variant affecting a canonical splice site and predicted to disrupt correct splicing of the *SATB2* transcript, also likely leading to *SATB2* haploinsufficiency. We thus consider the variants in these 16 individuals to be clear loss‐of‐function variants. In addition, four individuals had a missense variant in *SATB2*, two individuals had a 2q33.1 microdeletion and one individual had a mosaic frameshift variant.

Within the group of individuals with a missense variant (*n* = 4), three individuals were classified as primarily verbal (75%) and one individual was primarily nonverbal (25%). In the group of individuals with a loss‐of‐function single nucleotide variant (*n* = 16), six individuals were classified as primarily verbal (37.5%) and 10 individuals as primarily nonverbal (62.5%). In the group of individuals with a missense variant, the age equivalents of Vineland adaptive functioning total scores ranged from 36 to 59 months (median 39.5), while in the group of individuals with loss of‐function variants the range was 12 to 44 months (median 29).

## 5 DISCUSSION

With this study, we aimed to delineate oral motor, speech, language profiles in the context of cognitive and adaptive functioning in 23 individuals with *SATB2*‐associated syndrome, a neurodevelopmental disorder generally characterized by intellectual disability and prominent speech and language problems. We used standardized observations and questionnaires and validated tests to characterize speech/language and oral motor functioning and neuropsychological capacities of primarily verbal (*n* = 11, 47%) and primarily nonverbal (*n* = 12, 52%) individuals with SAS.

Regarding oral motor functioning, almost all individuals (87%) were reported to have feeding problems in addition to speech problems. In the nonverbal group, oropharyngeal problems (chewing with choking) were common (75%) and in the verbal group a milder phenotype with mainly oral phase problems with chewing and/or overstuffing was seen (87.5%). This finding is in contrast to a recently published study on speech, language and feeding phenotypes in SAS, which reported pharyngeal phase problems in the majority of assessed individuals,^5^ both nonverbal and verbal. Differentiating between underlying mechanisms causing dysphagia in individuals with SAS is important for therapeutic management decisions, as oral phase problems generally require different approaches than pharyngeal phase problems. In addition to dysphagia, about half of the children in our cohort (48%) suffer from drooling, a problem more present in the nonverbal group. All in all, oral motor problems seem to be a significant problem in the nonverbal group, suggesting that personalized approaches are needed to evaluate and treat oral motor and feeding difficulties.

Using standardized language tests, expressive and receptive language deficits were found in all individuals that could be assessed for such abilities. Almost all had severe receptive language delays, but further discrimination of the individual levels was hampered by floor effects reached using these tests. Age equivalents of receptive language scores correspond to age equivalents of total Vineland scores, suggesting that the Vineland screener is a useful instrument to give an indication of receptive language in clinical practice, with further studies needed to gain insight in the underlying (shared) theoretical constructs. In 10/11 verbal individuals, differentiation of speech symptoms led to diagnoses of speech‐related disorders. While childhood apraxia of speech was most common, other diagnoses included phonological delays, dysarthria and articulation impairment. For *SATB2‐*associated disorder, a previous study reported a diagnosis of childhood apraxia of speech in all 40 individuals with enough verbal ability in their SAS cohort.^5^ While one might hypothesize that individuals with the same genetic disorder have similar speech and language phenotypes, the results of our detailed diagnostic speech profiling show that even with the same genetic syndrome, divergent speech problems may occur. Subgroups with childhood apraxia of speech, phonological delay, dysarthria and articulation impairment are thought to represent different underlying deficits,^28^ although such problems might sometimes co‐occur. These underlying deficits can be described in terms of problems with phonological encoding, speech motor planning, speech motor programming and speech motor execution.^29^ The results of the current study show the need for detailed personalized speech and language assessments in each individual with SAS, since distinct speech problems will benefit from different approaches to intervention.

It is currently unclear which processes underly the absence of speech as a primary mode of communication in the nonverbal group. Based on the results of cognitive, language and oral motor assessments, we would expect these individuals to be able to develop a certain level of speech. As a result, the absence of speech is possibly the result of neurobiological mechanisms involved in the speech process, or behavioral characteristics, but is not simply secondary to cognition, language, or oral motor impairments. It is hard to generate hypotheses regarding the specific speech process involved, as we identified several different processes in the verbal group that contributed to impaired speech development. Possibly, the verbal versus nonverbal distinction in SAS is mainly caused by the severity of impairments in one or more of the speech processes. It is interesting to note in this context that observation of individuals during assessments showed limited levels of initiation of communication, in addition to lower levels of frustration than would be expected based on the severely limited communication in most individuals.

Generally, it is difficult to assess the IQ levels in individuals with ID by using conventional methods that are based on the normal population. Therefore, we converted the Wechsler based test scores in corresponding developmental age equivalents, in order to derive useful scores for all or several indices for a subset of individuals. Nonetheless, for the vast majority of the cohort we were able to obtain scores on adaptive functioning. Previous studies using Vineland Adaptive Behavior Scales have shown that different genetic disorders can give rise to distinctive profiles of adaptive functioning, which might also be partly age‐dependent.^30^, ^31^, ^32^ The relative weakness of communication in the adaptive functioning profile observed in the Vineland scores within our study is in line with the findings from direct speech and language assessments, as well as the literature on SAS so far.^33^ Variations in adaptive functioning domains with relatively strong daily‐living skills based on Vineland questionnaires are commonly reported in other neurodevelopmental disorders.^32^, ^34^, ^35^ As already shown for other genetic syndromes, the assumption that cognitive functioning is strictly related to all adaptive functioning domains does also not apply to SAS.^36^ Classifying an accurate level of ID based on the required equal weighting of intellectual functioning (i.e., IQ) and level of adaptive functioning^37^ is therefore a challenge and more in‐depth analysis of intellectual functioning and adaptive functioning is required.

In the literature on SAS, behavioral issues have been reported in the majority of individuals, with different forms of challenging behavior being present, including autistic traits, hyperactivity and aggression.^33^, ^38^ In our study, autistic traits were mentioned by parents or caregivers in only four individuals (none meeting requirements for a formal ASD diagnosis), and two individuals (9%) received methylphenidate because of attention problems. Broader behavioral issues in our study cohort were evaluated using CBCL/ABCL questionnaires, and we found one third of assessed individuals to have scores within the clinical range, which seems to be in line with the level of ID and/or verbal proficiency. Clinical range scores did not reflect the level of test cooperation. Growing literature on genetic syndromes from a multidisciplinary perspective (i.e., neuropsychology, psychiatry and clinical genetics) shows that particular behaviors should be interpreted in a wider context in order to understand if, and in what way, they should be regarded as specific to the phenotype.^39^, ^40^ Research in KBG syndrome, for instance, showed that social difficulties reported in affected individuals might well be related to (the level of) ID instead of reflecting a specific ASD trait.^41^ A longitudinal meta‐analysis by Chow et al.^42^ shows that receptive language skills in particular have a strong predictive property when it comes to challenging behavior, and that improving (receptive) language skills can have a mitigating effect on the development of behavioral problems. Although our results do not directly support this link between problems in language and behavior, it is possible that the relatively low levels of frustration observed in individuals in our study and a related lack of initiation contribute to the severe speech phenotype. Findings like these warrant a broad and strong dimensional approach to clinical assessment using gold‐standard instruments and a careful consideration of contextual factors to correctly interpret a particular behavior as part of the SAS phenotype profile.^39^, ^40^ Research has also shown that it is necessary to interpret challenging behavior in ID in relation to contextual variables, in order to establish an effective intervention plan.^43^

Different types of heterozygous *SATB2* disruptions were found in the individuals included in our study. While there is some evidence that missense variants of *SATB2* might be associated with milder phenotypes,^3^ functional characterization of effects of variants in this gene has so far been limited. It is therefore unclear whether missense variants have different effects from the loss‐of‐function that is assumed for most other variants.^3^, ^44^ As *SATB2* encodes a transcription factor that can have pleiotropic effects on multiple different pathways and developmental processes in the brain, it is important to realize that many different factors (e.g., stochastic developmental factors) might ultimately contribute to the phenotypic presentation, even between individuals with identical pathogenic variants.

In addition to individuals with single nucleotide variants affecting *SATB2*, our cohort included two individuals (individual 4 and 20) in which a de novo 2q33.1 microdeletion including the *SATB2* gene was reported. Re‐evaluation of the original array‐CGH report of individual 20 however could not confirm the involvement of the *SATB2* gene with certainty. We therefore performed a CytoScan XON array analysis, which showed that the deletion was located just six kilobases downstream of *SATB2*. Although the breakpoints of the deletion were located outside the coding region of *SATB2*, and thus a loss‐of‐function effect via haploinsufficiency is unlikely for this individual, positional effects of this deletion on *SATB2* gene expression cannot be excluded.

Our study has some limitations that should be taken into account. First, because of the low prevalence of *SATB2* variants in the population, it is not possible to study a large cohort of affected individuals with the same native language in the same age range. The consequent differences in chronological ages in our cohort, as well as the varying levels of cognitive functioning, made systematic testing using comparable tests more difficult and in some cases impossible, leading to suboptimal data collection. In line with this, to show true capacities of all individuals in this study, modifications to the standardized study assessments had to be made, which might have influenced the results obtained. Another limitation of our study methods is potential for examiner bias, as well as the possible bias caused by parental reporting. Lastly, it is unclear if the chronological age of individuals in this study might have affected the results, as current possibilities on diagnostics, speech therapy and education are very different compared with the situation decades ago. While these limitations are applicable for the study data on a group level, they do not apply for the usability of the data on an individual level, for example, intra‐individual comparisons in a longitudinal study setting.

Nonetheless, our research can serve as a base for future studies on speech, language, oral motor and cognitive functioning in SAS. Ideally, longitudinal studies should be executed in which children with a SAS diagnosis at a young age are included for early diagnostics on a speech, language and cognitive level, and for subsequent targeted interventions. In addition, for future studies we recommend the inclusion of nonverbal test batteries aimed at specific cognitive domains (i.e., attention, processing speed, executive functioning) as well as general level of intellectual functioning, combined with gold standard proxy instruments, to be able to better define cognitive performance, behavior profiles and adaptive functioning in individuals with SAS of all levels.

In summary, with this study we provide a delineation of speech, language and oral motor skills in individuals with SAS, combined with emerging data on neuropsychological functioning. While overlapping and highly recurrent features were seen for both the speech and language domain and the adaptive functioning profile, there was also a high variability observed, mainly in severity of features. This study can provide families, speech therapists, psychologists and other caregivers with the necessary information to guide diagnostic and treatment approaches in order to obtain the best functional outcomes in individuals with *SATB2* associated syndrome.

## CONFLICT OF INTEREST

The authors have no conflicts of interest to declare.

## Supporting information


**Table S1** Supporting information

## Data Availability

The data that support the findings of this study are available on request from the corresponding author. The data are not publicly available because of privacy or ethical restrictions.
